# Catalytic Redundancies and Conformational Plasticity Drives Selectivity and Promiscuity in Quorum Quenching Lactonases

**DOI:** 10.1021/jacsau.4c00404

**Published:** 2024-08-23

**Authors:** Marina Corbella, Joe Bravo, Andrey O. Demkiv, Ana Rita Calixto, Kitty Sompiyachoke, Celine Bergonzi, Alfie-Louise R. Brownless, Mikael H. Elias, Shina Caroline Lynn Kamerlin

**Affiliations:** † Departament de Química Inorgànica (Seeió de Química Orgànica) & Institut de Química Teòrica i Computacional (IQTCUB), Universitat de Barcelona, Martíi Franquès 1, 08028 Barcelona, Spain; ‡ Department of Chemistry − BMC, Uppsala University, BMC Box 576, S-751 23 Uppsala, Sweden; § BioTechnology Institute, University of Minnesota, Saint Paul, Minnesota 55108, United States; ∥ LAQV, REQUIMTE, Departamento de Química e Bioquímica, Faculdade de Ciências, Universidade do Porto, Rua do Campo Alegre, s/n, 4169-007 Porto, Portugal; ⊥ Department of Biochemistry, Molecular Biology and Biophysics, University of Minnesota, Saint Paul, Minnesota 55108, United States; # School of Chemistry and Biochemistry, Georgia Institute of Technology, 901 Atlantic Drive NW, Atlanta, Georgia 30332, United States

**Keywords:** quorum sensing, biofilm formation, lactonases, enzyme promiscuity, enzyme mechanism

## Abstract

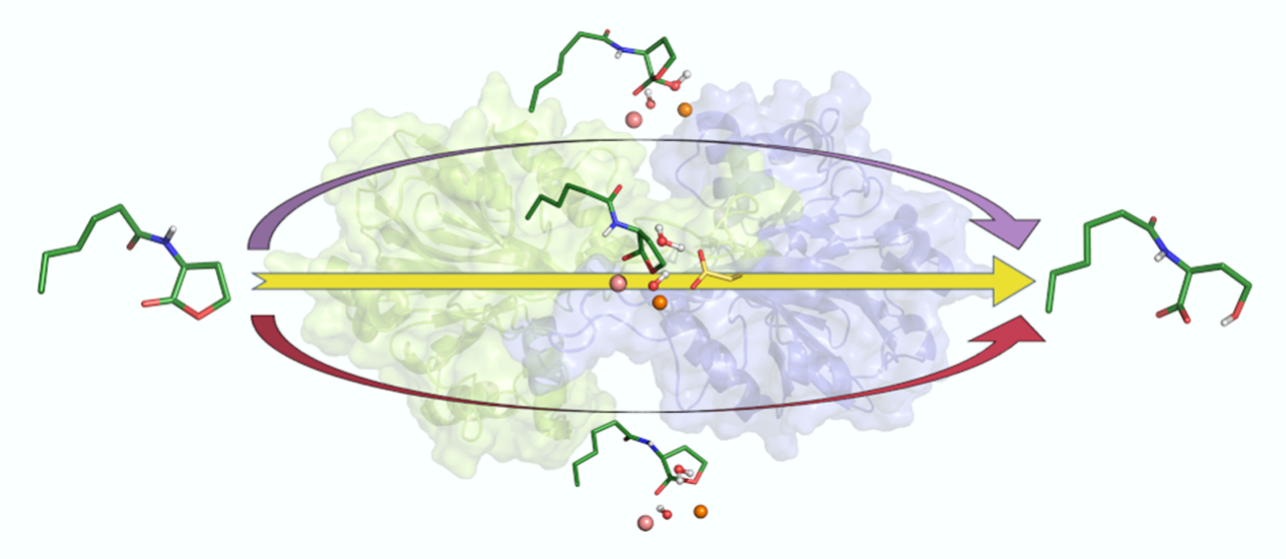

Several enzymes from the metallo-β-lactamase-like family of lactonases (MLLs) degrade *N-*acyl L-homoserine lactones (AHLs). They play a role in a microbial communication system known as quorum sensing, which contributes to pathogenicity and biofilm formation. Designing quorum quenching (*QQ*) enzymes that can interfere with this communication allows them to be used in a range of industrial and biomedical applications. However, tailoring these enzymes for specific communication signals requires a thorough understanding of their mechanisms and the physicochemical properties that determine their substrate specificities. We present here a detailed biochemical, computational, and structural study of GcL, which is a highly proficient and thermostable MLL with broad substrate specificity. We show that GcL not only accepts a broad range of substrates but also hydrolyzes these substrates through at least two different mechanisms. Further, the preferred mechanism appears to depend on both the substrate structure and/or the nature of the residues lining the active site. We demonstrate that other lactonases, such as AiiA and AaL, show similar mechanistic promiscuity, suggesting that this is a shared feature among MLLs. Mechanistic promiscuity has been seen previously in the lactonase/paraoxonase PON1, as well as with protein tyrosine phosphatases that operate via a dual general acid mechanism. The apparent prevalence of this phenomenon is significant from both a biochemical and protein engineering perspective: in addition to optimizing for specific substrates, it may be possible to optimize for specific mechanisms, opening new doors not just for the design of novel quorum quenching enzymes but also of other mechanistically promiscuous enzymes.

## Introduction

The molecular determinants responsible for the high proficiency and specificity of enzymes are often discussed. However, while the chemical role of key active site and catalytic residues are typically depicted as uniquely specific, recent examples of enzymatic promiscuity suggest that the same active site residues may perform different roles in the same enzyme, allowing for both substrate and catalytic promiscuity.^1−4^ On one hand, different subsets of active site residues in a large binding pocket can be used to facilitate reactivity with different substrates.^4^ Conversely, those same active site residues may also be capable of performing multiple tasks within the same active site to catalyze the same reaction, when the preorganization of reactive residues allows for several, energetically close, reaction trajectories. These two scenarios are not mutually exclusive. For example, both scenarios have been observed with the enzyme serum paraoxonase 1 (PON1), a catalytically promiscuous organophosphatase/lactonase.^4,5^ In this work, we focus on providing a detailed structural and mechanistic description of the multifunctional role of active site residues in different members of a catalytically and mechanistically promiscuous family of enzymes.

Our work discusses lactonases (EC 3.1.1.81) from the metallo-β-lactamase-like family of lactonases (MLLs). MLLs degrade *N*-acyl-L-homoserine lactones (AHLs), molecules that are used in a microbial communication system called quorum sensing (QS) to coordinate a variety of behaviors, including virulence and biofilm formation.^6,7^ By degrading AHLs, these enzymes can interfere with microbial signaling and are therefore called quorum quenchers (*QQ*). They have been reported to inhibit behaviors that are regulated by bacterial QS such as virulence factor production and biofilm formation, and can also alter microbiome population structure.^8−13^ As a result, the mechanisms of *QQ* enzymes, as well as their engineering for targeted biotechnological applications (including optimizing their activity and their stability), are currently topics of intensive research. Further, *QQ* enzymes and formulations with these enzymes can prevent biofouling and biocorrosion and are promising candidates for biomedical applications.^10,14−18^

Lactonases have been identified from a wide range of organisms, including archaea, bacteria, fungi, and mammals.^18−25^ Three main families of lactonases have been identified,^20^ all of which are metalloenzymes.^26−28^ Paraoxonases (PONs), primarily isolated from mammals, exhibit a six-bladed β-propeller fold, and a monometallic (calcium) active site center.^20^ Phosphotriesterase-like lactonases (PLLs) exhibit an (α/β)_8_-fold and a bimetallic active site center.^29^ Metallo-β-lactamase-like lactonases (MLLs) possess an αββα-fold and a conserved dinuclear metal-binding motif, HxHxDH, involved in the coordination of the bimetallic active site center and are the focus of this work. Numerous representative enzymes from this family have been kinetically and/or structurally characterized, including AiiA,^30^ AiiB,^31^ AidC,^32^ MomL,^33^ and AaL.^34^

**Figure 1:**
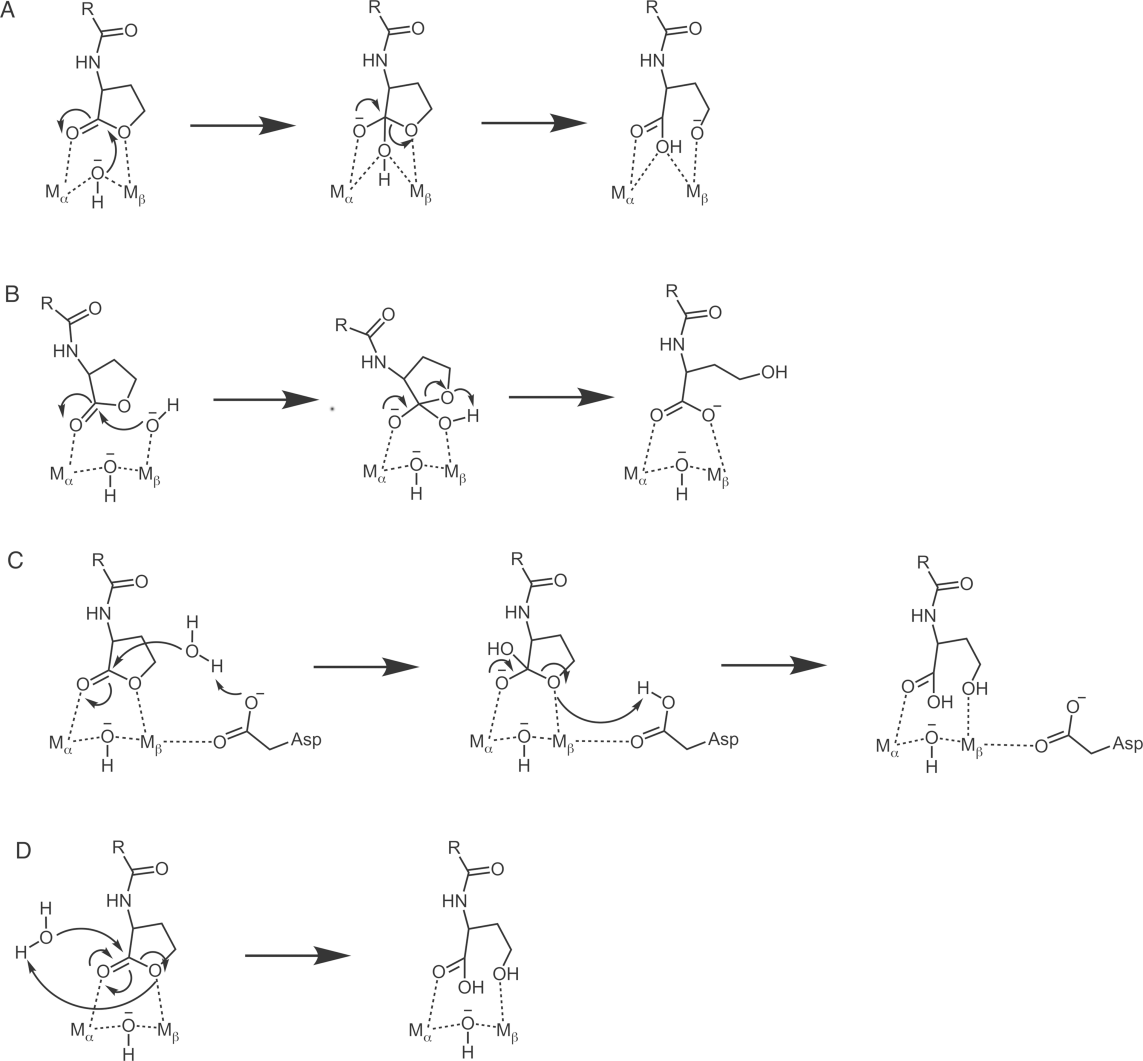
Plausible mechanisms for the hydrolysis of *N*-acyl-L-homoserine lactones by lactonases. (A) Stepwise nucleophilic attack on the carbonyl carbon of the lactone ring by the bridging hydroxide ion followed by breakdown of the resulting tetrahedral intermediate (“bridging hydroxide mechanism”). (B) Stepwise nucleophilic attack on the carbonyl carbon of the lactone ring by a terminal hydroxide ion followed by the breakdown of the resulting tetrahedral intermediate (“terminal hydroxide mechanism”). (C) Stepwise general base catalyzed mechanism in which the side chain of a metal-bound aspartic acid (Asp122 using GcL numbering) acts as a general base to activate the nucleophilic water molecule followed by the breakdown of the resulting tetrahedral intermediate (“Asp mechanism”). (D) Concerted nucleophilic attack on the carbonyl carbon of the lactone ring by an active site water molecule activated by proton transfer to the lactone ring oxygen and opening of the lactone ring (“concerted mechanism”). Note that, for simplicity, we have shown the ring oxygen in the product state of mechanisms A and B to be deprotonated; however, the ring-opening reaction would benefit from protonation by an acid catalyst, the precise identity of which can vary depending on the system. The shorthand designations for each mechanism, shown in parentheses, will be used throughout the text.

Understanding the mechanism and selectivity of these enzymes toward specific lactones would facilitate more efficient engineering of lactonases for biotechnological applications through approaches such as the construction of focused libraries for directed evolution. This is particularly important as the selectivity of these enzymes toward different substrates is complex and can depend on, for example, the acyl chain lengths of the different AHLs.^30,33,35,36^ In addition, the chemical structure of the lactone can control the specificity of cell signaling.^37−39^ Despite the importance of this question, the catalytic mechanism of lactonases (Figure 1) is not yet fully understood. In MLLs and PLLs, the bimetallic center is hypothesized to activate the substrate and a catalytic water molecule.^20^ The nature of this nucleophilic water molecule is yet unclear: while the nucleophile is often hypothesized to be the metal-bridging water molecule in the form of a hydroxide ion (Figure 1A),^20^ compelling evidence for this mechanism has been elusive due to the difficulty of isolating transition states in crystal structures.

A near-identical mechanism to that shown for lactone hydrolysis in Figure 1A (“bridging hydroxide mechanism”) has been proposed for organophosphate hydrolysis by phosphotriesterases (PTEs),^40−42^ a closely related enzyme family to PLLs. PTEs possess a similar bimetallic active site; however, recent experimental evidence has suggested that rather than the bridging hydroxide ion, the nucleophile is more likely to be a terminal hydroxide ion (Figure 1B, “terminal hydroxide mechanism”).^43^ This is in agreement with data from studies of designed binuclear catalysts of phosphate hydrolysis reactions,^44^ computational studies of a related enzyme, methyl parathion hydrolase,^45^ as well as other metallophosphatases.^46,47^ We note again here that a terminal hydroxide ion would be expected to have a more favorable p*K*_a_ for nucleophilic attack than the bridging hydroxide ion, the p*K*_a_ of which would be substantially depressed by coordination to two metal ions (in the range of 9–10 for the terminal hydroxide ion,^48−50^ depending on metal ion, compared to 7.3 for the bridging hydroxide ion in the case of the analogous enzyme phosphotriesterase^51^). The terminal hydroxide ion would also have more structural flexibility than the bridging hydroxide ion, which is held tightly in place by the two metal ions it coordinates. Moreover, it is possible that the nucleophile is not metal coordinated at all, but rather is an active site water molecule activated, for example, by general base catalysis through a metal-bound aspartic acid in the active site, e.g., Asp122 (Figure 1C, “Asp mechanism”), or by concerted proton transfer to the lactone ring oxygen (Figure 1D, “concerted mechanism”).

Additionally, the hydrolysis of the lactone ring involves a leaving alcoholate group. This poor leaving group may benefit from protonation by an acid catalyst. It has been proposed that this protonation is carried out by a metal-coordinating aspartic acid residue in the case of the lactonase AiiA.^30^ In a mechanism such as that shown in Figure 1C, the protonated aspartic acid that is generated upon nucleophilic attack to open the lactone ring could then act as a general acid to assist in leaving group departure. When taking into account the potentially short lifetime of the tetrahedral intermediate formed upon lactone ring opening (i.e., the reaction can potentially proceed via a borderline mechanism that is almost concerted in nature^52^), then the number of potential viable mechanisms becomes a large combinational problem, and distinguishing between these different mechanisms does not seem experimentally possible. The full span of potential mechanisms has not yet been considered for either lactonases or organophosphate hydrolases with analogous active sites. Simulation approaches are ideal to sample these different mechanisms as they allow for the direct comparison of different reaction pathways with a range of lactone substrates containing different acyl tail lengths. Obtaining deeper insight into possible mechanisms across a range of substrates will also provide insight into plausible catalytic mechanisms for other metallohydrolases that have similar active site architectures.

Here we aim to resolve the catalytic mechanism(s) of lactonases from the metallo-β-lactamase superfamily. We provide the structural and biochemical analysis of the lactonase GcL (WP_017434252.1), isolated from the thermophilic bacteria *Parageobacillus caldoxylosilyticus*.^36,53^ GcL is a thermostable, highly proficient lactonase with broad substrate specificity (*k*_cat_/*K*_M_ values range between 10^4^ to 10^6^ M^–1^ s^–1^) for substrates such as *N-*butyryl (C4) L-homoserine lactone (HSL) and *N*-decanoyl (C10)-HSL. We combine unique structural data, including the structure of GcL in complex with an intact substrate (*N*-hexanoyl-HSL (C6-HSL)) and a hydrolytic product (hydrolysis of *N*-octanoyl-HSL (C8-HSL)), with empirical valence bond (EVB) simulations^54^ to probe possible catalytic mechanisms for lactone hydrolysis. The results of computational modeling were tested against mutational data in GcL and extended to other lactonases from the metallo-β-lactamase superfamily, including AaL from the moderately thermophilic bacterium *Alicycloacter acidoterrestis*,^55^ and the more distantly related AiiA, from the mesophilic bacterium *Bacillus thuringiensis*.^30,56^ Results reveal that the enzymatic reaction can proceed via (at least) two chemically distinct but energetically similar mechanisms, with the precise pathway taken being dependent on the specific substrate or enzyme variant. This catalytic versatility, making use of a distinct subset of active site residues, is consistent with the reported enzymatic promiscuity and broad selectivities of lactonases.

Mechanistic promiscuity has been proposed for an analogous lactonase, PON1, which was suggested to both possess catalytic backups in the active site,^4^ as well as have more than one simultaneously viable mechanism.^5^ Similarly, several protein tyrosine phosphatases also appear to operate via a dual general acid mechanism.^57−59^ Such mechanistic promiscuity has not been observed in the literature as a widespread phenomenon; however, the data presented here suggest that it is, at minimum, common to multiple distinct quorum quenching lactonases. This is significant not only from a biochemical standpoint but also from an engineering perspective: protein engineering efforts often focus on optimizing activity for specific substrates and, while doing so, can also optimize for a specific *reaction mechanism* out of a pool of viable mechanisms.^5^ Here, we extend the potential generalizability of this concept across several MLL enzymes. This opens the door to new strategies in the design and engineering of not just biotechnologically important *QQ* enzymes, but also other enzymes that may be mechanistically promiscuous.

## Results and Discussion

### The Structure of GcL in Complex with L-Homoserine Lactone

**Figure 2:**
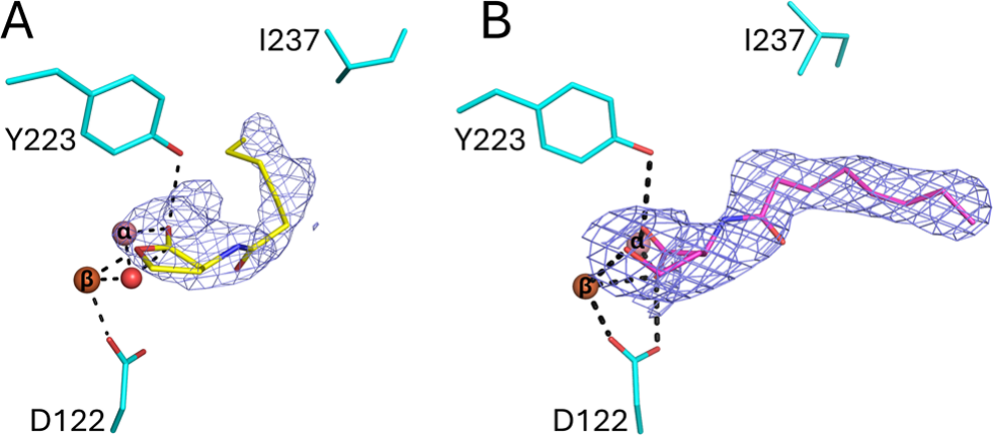
Structures of GcL structures in complex with lactone substrate and hydrolysis products. (A) The active site of GcL (cyan sticks) bound to its substrate C6-HSL (9AYT; yellow sticks, modeled at 0.8 occupancy). The lactone ring sits on the bimetallic active site (pink and orange spheres). (B) The structure of GcL in complex with a product of the hydrolysis of C8-HSL (pink sticks; PDB ID: 9B2O; modeled at 0.7 occupancy). The mesh shows the *F*_o_–*F*_c_ omit maps contoured at 2.2 σ. Metal cations are shown as pink (cobalt, α) and orange (iron, β) spheres, reduced size for clarity.

The structure of GcL was previously determined and described in refs (36,55). GcL possesses a bimetallic active site center containing iron and cobalt cations. The binuclear center, common to all MLLs, is coordinated by five histidine residues and two aspartic acid residues.^20^ A water molecule bridges both metal cations, which has previously been hypothesized to be the reaction nucleophile in this family.^20^ The structure of GcL bound to C6-HSL (0.8 occupancy) is overall similar to the previously obtained structures of GcL bound to the substrates C4- and 3-oxo-dodecanoyl (3-oxo-C12)-HSL^36^ (Figure S1). The lactone ring of the substrate sits on the bimetallic active site, with the carbonyl oxygen atom of the lactone ring interacting with the cobalt cation (2.6 Å) and the ester oxygen atom interacting with the iron cation (2.2 Å). In combination, these interactions likely increase the electrophilic character of the carbonyl carbon atom. The carbonyl oxygen atom is also hydrogen bonded to the hydroxyl group of Tyr223 (3.0 Å). The *N-*alkyl chain of C6-HSL is kinked and interacts with residue Ile237 (Figure 2).

### Structure of GcL in Complex with the L-Homoserine Lactone Hydrolysis Products

We solved the structure of GcL in complex with AHL-hydrolytic products bound to the active site through cocrystallization with C8-HSL (Table S1). Attempts to cocrystallize GcL with C6-HSL resulted in crystals with very low ligand occupancy (∼0.5, not shown). While the model of GcL in complex with the hydrolytic product of C8-HSL (Figures 2 and S2) is limited in its accuracy by a relatively low ligand occupancy (∼0.7), it reveals that the negative charge of the hydrolytic product carboxylate group is stabilized by the bimetallic active site center, and that the alcohol group interacts with the α-metal (iron; 2.7 Å; monomer L).

In addition, the carboxylate group of the C8-HSL product is positioned between the two metal cations, 2 Å from the cobalt cation and 2.8 Å from the iron cation (Figure 2B; monomer L). The other oxygen atom of the carboxylate is hydrogen bonded to the hydroxyl group of Tyr223 (2.7 Å). During lactone hydrolysis, the protonation of the leaving alcoholate group may be an important, catalytically limiting step. The position of the alcohol group is 3.3 and 3.5 Å from Asp122 and Tyr223, respectively. This suggests that the side chains of Tyr223 and Asp122 are possible acid catalysts for the reaction, although a protonated Asp122 side chain (protonated in the first reaction step, Figure 1) would be expected to have a more favorable p*K*_a_ to fulfill the role of an acid catalyst. An alternative hypothesis, also considered here (Figure 1), would be an intramolecular protonation mechanism. These mechanisms are very similar: they all involve proton transfer from either an amino acid side chain or a water molecule, making it very hard to distinguish between them experimentally.

### Probing the Roles of Acid Catalyst Candidates Tyr223 and Asp122

Tyr223 and Asp122 are the only polar residues in the vicinity of the substrate in the active site of GcL. Remarkably, the presence of a tyrosine residue side chain is a conserved feature of lactonases, including those from different folds.^60^ Asp122 is also conserved and is involved in coordinating the metal cation. The corresponding Asp122 residue in the lactonase AiiA was previously hypothesized to protonate the leaving group in a structural and mutagenesis study.^30,56^ To investigate the importance of these residues, we therefore substituted Tyr223 with phenylalanine and Asp122 with asparagine in GcL, eliminating their role in acid catalysis, and kinetically characterized the resulting GcL variants (Table 1 and Figures S3 and S4).

**Table 1:** Kinetic Parameters for the Hydrolysis of AHL Substrates and the Phosphotriester Paraoxon by Wild-Type GcL and Variants^a^

enzyme	substrate	*k*_cat_ (s^–1^)	fold Δ to WT	*K*_M_ (μM)	fold Δ to WT	*k*_cat_/*K*_M_ (s^–1^ M^–1^)	fold Δ to WT
WT^b^	C4	19.06 ± 1.51	-	229 ± 57	-	8.3 (±2.2) × 10^4^	-
	C6	8.95 ± 0.48	-	7.97 ± 1.89	-	1.1 (±0.3) × 10^6^	-
	C8	1.29 ± 0.04	-	3.12 ± 0.57	-	4.1 (±1.0) × 10^5^	-
	C10	5.48 ± 0.37	-	1.45 ± 0.47	-	3.8 (±1.3) × 10^6^	-
	Paraoxon	ND	-	ND	-	3.1 (±0.2) × 10^1^	-
D122N	C4	1.74 ± 0.10	11.0↓	41.0 ± 9.14	5.6↓	4.25 (±0.98) × 10^4^	2.0↓
	C6	2.60 ± 0.24	3.4↓	13.0 ± 6.16	1.6↑	2.00 (±0.96) × 10^5^	5.5↓
	C8	4.45 ± 0.40	3.4↑	4.36 ± 1.69	1.4↑	1.02 (±0.41) × 10^6^	2.5↑
	C10	3.19 ± 0.16	1.7↓	0.52 ± 0.18	2.8↓	6.08 (±2.12) × 10^6^	1.6↑
	Paraoxon	ND	-	ND	-	5.79 (±0.45) × 10^1^	1.9↑
Y223F	C4	0.79 ± 0.15	24.1↓	758 ± 275	3.3↑	1.04 (±0.43) × 10^3^	79.7↓
	C6	5.01 ± 0.46	1.8↓	92.7 ± 36.7	11.6↑	5.41 (±2.19) × 10^4^	20.3↓
	C8	ND	-	ND	-	1.66 (±0.08) × 10^3^	308↓
	C10	1.07 ± 0.16	5.1↓	523 ± 167	361↑	2.04 (±0.72) × 10^3^	1870↓
	Paraoxon	ND	-	ND	-	3.34 (±0.16) × 10^2^	10.8↑
A157G	C4	63.89 ± 6.66	3.4↑	1144 ± 192	5.0↑	5.58 (±1.05) × 10^4^	1.5↓
	C6	9.93 ± 1.08	1.1↑	135 ± 33	16.9↑	7.37 (±1.97) × 10^4^	14.9↓
	C8	7.97 ± 0.33	6.2↑	19.34 ± 3.28	6.2↑	4.12 (±0.72) × 10^5^	1.0
	C10	3.62 ± 0.23	1.5↓	3.27 ± 1.08	2.3↑	1.11 (±0.37) × 10^6^	3.4↓
	Paraoxon	ND	-	ND	-	7.7 ± 0.3 × 10^1^	2.5↑
A157S	C4	24.9 ± 2.52	1.3↑	475 ± 103	2.1↑	5.24 (±1.25) × 10^4^	1.6↓
	C6	2.58 ± 0.20	3.5↑	15.6 ± 4.95	2.0↑	1.65 (±0.54) × 10^5^	6.7↓
	C8	4.44 ± 0.42	3.4↑	25.11 ± 9.27	8.0↑	1.77 (±0.67) × 10^5^	2.3↓
	C10	3.03 ± 0.31	1.8↓	13.66 ± 5.19	9.4↑	2.22 (±0.87) × 10^5^	17.1↓
	Paraoxon	0.14 ± 0.008	-	964 ± 136	-	1.42 ± 0.22 × 10^2^	4.6↑
I237M	C4	9.22 ± 1.00	2.0↓	218 ± 64	-	4.23 (±1.33) × 10^4^	2.0 ↓
	C6	0.95 ± 0.04	9.4↓	2.38 ± 0.68	3.3↓	3.99 (±1.15) × 10^5^	2.8↓
	C8	1.64 ± 0.06	1.3↑	2.25 ± 0.54	1.6↓	7.28 (±1.76) × 10^5^	1.8↑
	C10	1.10 ± 0.07	5.1↓	7.62 ± 2.14	5.3↑	1.44 (±0.42) × 10^5^	26↓
	Paraoxon	0.05 ± 0.001	-	1341 ± 354	-	3.73 ± 1.11 × 10^1^	1.2↑
G156P	C4	8.72 ± 0.69	2.2↓	771 ± 140	3.4↑	1.13 (±0.22) × 10^4^	7.3↓
	C6	2.44 ± 0.31	3.7↓	563 ± 150	70.7↑	4.34 (±1.28) × 10^3^	254↓
	C8	1.44 ± 0.09	1.1↑	8.22 ± 2.92	2.6↑	1.75 (±0.63) × 10^5^	2.3↓
	C10	0.73 ± 0.02	7.5↓	1.31 ± 0.34	1.1↓	5.60 (±1.45) × 10^5^	6.8↓
	Paraoxon	ND	-	ND	-	1.80 ± 0.1 × 10^2^	5.8↑

a Data were measured at pH 8.3 and 25 °C. Initial velocities were fitted to the Michaelis–Menten equation using GraphPad Prism 5 for Windows (GraphPad Software, San Diego, California) to obtain the catalytic parameters (Figures S3–S8). Replicates with technical errors (e.g., pipetting errors or failed) were excluded from the Michaelis–Menten analysis.

b WT catalytic parameters were obtained from ref (36). ND: not determined. Linear regression was used for fitting because saturation could not be reached.

Kinetic characterization of these two variants reveals that the Asp122Asn substitution causes a reduction in catalytic efficiency against C4- and C6-HSL (2- and ∼6-fold, respectively), however, catalytic efficiencies against longer chain AHLs are slightly increased. (Table 1). The reduction in catalytic efficiency against C4- and C6-HSL effect is smaller than those observed for the corresponding mutation made to AiiA (D108N; ∼36-fold reduction of catalytic efficiency with C6-HSL and Co^2+^, see ref (30)). Surprisingly the mutation does not abolish lactonase activity for either enzyme. The alterations in GcL activity may still suggest that this residue is important, but the interpretation of this effect is complicated by the clear role of this residue in metal coordination. This could be seen through crystallization, where multiple conformations were captured: one where both metals are present at high occupancy, exhibiting an active site configuration very similar to that of the wild-type enzyme (Figure S9); and a second form where the active site shows no bound β-metal (or bound with very low occupancy) (Figure S9). This substitution may destabilize the bimetallic center, possibly by decreasing the affinity of the β-site for metals.

Conversely, Tyr223 is not involved in metal cation coordination, yet the Tyr223Phe substitution consistently significantly reduces the lactonase activity of GcL for all tested AHLs (>2 orders of magnitude reduction in catalytic efficiency for C8- and C10-HSL; Table 1). This effect is in the range of the changes described for the equivalent mutation performed in AiiA (Y194F; ∼169-fold reduction of catalytic efficiency with C6-HSL and Co^2+^, see ref (30)). In addition, the evaluation of the paraoxonase activity, i.e. the promiscuous ability of GcL to hydrolyze this phosphotriester, shows that the Tyr223Phe substitution only impairs lactonase activity: the variant exhibits a paraoxonase activity ∼11-fold higher than that of the wild-type enzyme. This observation, where the same substitution has opposing effects on alternative lactonase and phosphotriesterase activities, is not necessarily surprising and has been observed in our prior work on an analogous enzyme, serum paraoxonase 1 (PON1).^61,62^ While the impact of this substitution on *k*_cat_/*K*_M_ for the lactonase activity is significant, the impact on the turnover number, *k*_cat_, is much smaller (Table 1), suggesting that the tyrosine side chain is more likely to play an important role in substrate binding or positioning during the chemical reaction, as illustrated by its impact on the *K*_M_ values for AHL substrates. Overall, these mutagenesis data suggest that Tyr223 plays an important role in catalysis.

The previously elucidated GcL structure allowed the identification of other key residues lining the active site binding cleft that may be involved in substrate binding.^36^ Substituting the residues at these positions produced some variants with changes in the kinetic properties. We report here the kinetic characterization of several variants, including Ala157Gly, Ala157Ser, Gly156Pro and Ile237Met (Table 1 and Figure S10). While some of these variants show only modest changes in lactonase activity compared to the wild-type for several AHL substrates (e.g., Ile237Met), they show altered kinetics for specific substrates, resulting in changes in substrate preference. For example, the Ala157Ser variant exhibits a 6.7-fold and 17.1-fold reduction in catalytic efficiency against C6- and C10-HSL, respectively. The Gly156Pro variant exhibits larger changes in preference, with a 254-fold drop in catalytic efficiency against C6-HSL. Intriguingly, the decrease in substrate preference does not change linearly with the increase in chain length but shifts 2.3 and 6.8-fold for C8- and C10-HSL, respectively. This suggests that subtle substrate conformational sampling may occur differentially as a function of chain length (and, by extension, as a function of the acyl chain hydrophobic character and entropy).

To gain some molecular insights into the effects of these substitutions on the active site configuration, structures were solved for both Gly156Pro and Ile237Met variants. The structure of the Gly156Pro variant reveals that the Asn152-Ala157 loop is significant in accommodating long chain AHL substrates (Figure S11). The conformational change in this loop may be responsible for the altered substrate preference of the enzyme. On the other hand, the Ile237Met variant shows a slightly altered conformation of the Pro234-Asp240 loop, in the vicinity of the amide group of the AHL substrate (Figure S12). This conformational change is more distant from the atoms of a long acyl chain AHL substrate, and this is, therefore, consistent with the minimal changes in catalytic properties recorded for this variant.

### Empirical Valence Bond Simulations of the Hydrolysis of C6-HSL by Wild-Type GcL

As shown in Figure 1, lactone hydrolysis by GcL (and other lactonases) can proceed through multiple pathways that are difficult-to-impossible to distinguish between experimentally. As shown in Figure 1, these mechanisms can be either stepwise or concerted in nature. They can also involve either a metal-bound bridging or terminal hydroxide ion or a free water molecule as the nucleophile and, in the case of the Asp mechanism (Figure 1), can recruit an active site side chain (Asp122) to act as a general base.

**Figure 3:**
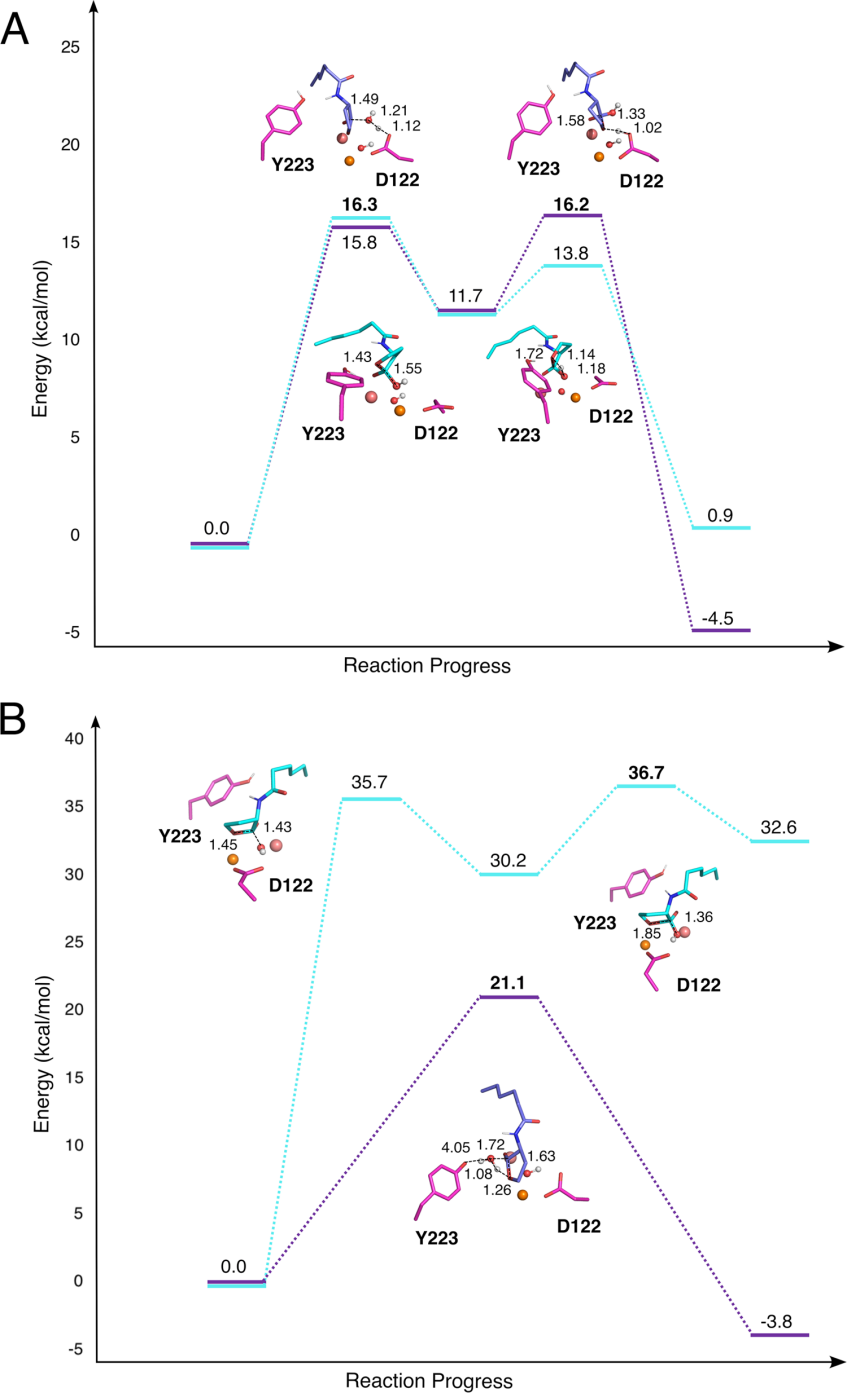
Representative structures of the transition states along the reaction coordinate for the hydrolysis of C6-HSL catalyzed by wild-type GcL via (A) the energetically favorable terminal hydroxide (cyan) and Asp (purple) mechanisms (Figure 1B,C and Table 2), and (B) the energetically unfavorable bridging hydroxide (cyan) and concerted (purple) mechanisms, as obtained from empirical valence bond simulations of these reactions. The structures shown here are the centroids of the top-ranked cluster obtained from clustering on root-mean-square deviation (RMSD), performed as described in Materials and Methods section. The distances labeled on this figure (Å) are averages at each transition state over all the EVB trajectories (see Table S2, with the corresponding data for the nonenzymatic reaction shown in Table S3, and metal–metal distances shown in Table S4). Shown here are the substrate, nucleophilic water, bridging hydroxide, Fe^2+^ (brown), Co^2+^ (salmon), and key catalytic residues. The remainder of the protein was omitted for clarity. The corresponding structures along the whole reaction coordinate for the four mechanisms are shown in Figures S13 and S14.

As our starting point, we constructed EVB models for the hydrolysis of C6-HSL by wild-type GcL through any of four possible reaction mechanisms: a bridging hydroxide mechanism (Figure 1A), a terminal hydroxide mechanism (Figure 1B), an Asp mechanism (Figure 1C), and a concerted mechanism (Figure 1D). Simulations were initiated from the crystal structure of wild-type GcL in complex with C6-HSL (PDB ID:9AYT, this study), as described in Materials and Methods. The results of these simulations are summarized in Figures 3, S13, and S14, and Table 2.

**Table 2:** Comparison of Calculated Activation Free Energies (kcal mol^–1^) for the Hydrolysis of C6-HSL Catalyzed by Wild-Type GcL through Different Mechanisms Considered in This Work, Compared to an Experimental Value of 16.2 kcal mol^–1^^a^

Mechanism	Δ*G*_1_^‡^	Δ*G*_int_	Δ*G*_2_^‡^	Δ*G*^0^
Bridging Hydroxide	35.7 ± 0.5	30.2 ± 0.8	**36.7 ± 1.1**	32.6 ± 1.2
Terminal Hydroxide	**16.3 ± 0.5**	11.7 ± 0.6	13.8 ± 0.8	0.9 ± 1.1
Asp	15.8 ± 0.2	11.7 ± 0.5	**16.2 ± 0.8**	–4.5 ± 0.7
Concerted	**21.1 ± 0.4**			–3.8 ± 0.5

a The different mechanisms considered here are summarized in Figure 1 of the main text. Δ*G*_1_^‡^, Δ*G*_int_, Δ*G*_2_^‡^, and Δ*G*^0^ correspond to the activation and free energies of the reaction for the formation of the transition states for nucleophilic attack on the lactone (Δ*G*_1_^‡^), the formation of the tetrahedral intermediate following nucleophilic attack (Δ*G*_int_), the transition state for the ring-opening reaction with breakdown of the intermediate (Δ*G*_2_^‡^), and the free energy for formation of the enzyme–product complex (i.e., the free energy of the reaction, Δ*G*^0^), except in the case of the concerted mechanism, which is modeled as a single-step reaction as described in the Materials and Methods section. All values shown here are averages and standard error of the mean over 30 independent EVB trajectories. The experimental turnover number (*k*_cat,_Table 1) at 25 °C is 8.95 ± 0.48 s^–1^, corresponding to an activation free energy of 16.2 kcal mol^–1^. Activation free energies for the rate-determining step of energetically favorable pathways are highlighted in bold. Note that, in the case of the terminal hydroxide mechanism, a 2.6 kcal mol^–1^ correction has been added to the energies of all steps to take into account the energetic cost of generating a metal-bound hydroxide nucleophile, as described in the Supporting Information.

Based on our simulations, we obtain very high activation free energies for the bridging hydroxide mechanism (Figure 1A, Table 2). This reaction pathway involves the loss of the electrostatically favorable metal–hydroxide interaction, as the charge migrates away from the metal ion, resulting in the high activation free energy presented in Table 2. We note that nucleophilic attack by the bridging hydroxide on paraoxon has been suggested to be energetically viable based on density functional theory (DFT)-based QM cluster or QM/MM calculations in other systems with similar active sites to GcL.^41,42,63^ However, interpretation of this data is complicated first by the fact that DFT calculations involving hydroxide as a nucleophile tend to significantly underestimate the activation free energies involved,^64−69^ a problem that is likely to be further exacerbated by the presence of the binuclear metal center in the active site, and secondarily by the issue that no alternate mechanisms were considered in these studies.

In contrast, both the terminal hydroxide and Asp mechanisms (Figure 1) appear to be energetically favorable and within a reasonable range of the upper limit of 16.2 kcal mol^–1^ for the experimental value (derived from the turnover number, *k*_cat_, Table 1). We note that our EVB simulations provide essentially indistinguishable activation free energies for these two mechanisms. This is plausibly due to the fact that both pathways proceed through nucleophilic attack by a similar hydroxide ion, with the main difference being in how the hydroxide ion is generated (lowering the p*K*_a_ of a metal-bound water molecule or aspartic acid as a base deprotonating the nucleophilic water molecule). In the case of the terminal hydroxide mechanism, this reaction follows a stepwise pathway, involving nucleophilic attack of a terminal hydroxide bound to the β-metal ion on the lactone ring with monodentate coordination to the α-metal ion through the C=O bond with intramolecular protonation of the intermediate concomitant to ring opening (Figure 1). The initial conformation of the lactone necessary to facilitate nucleophilic attack of a terminal hydroxide ion is slightly distorted compared with the putative structure from the crystal structure (Figure S15). However, the large active site of GcL could potentially accommodate multiple substrate binding modes. Furthermore, a similar pathway involving a terminal hydroxide ion has been suggested based on both experimental and computational data for a range of analogous systems.^44−47^

In the Asp mechanism (Figure 1C), the Asp122 side chain participates in acid–base catalysis during the reaction, first deprotonating the attacking nucleophile and then subsequently protonating the leaving group. A similar mechanism involving a metal-bound aspartic acid side chain has been suggested as a catalytic backup in an analogous lactonase, PON1,^4,5^ and, by extension, the existence of backup mechanisms is likely to be evolutionarily beneficial in scavenger enzymes. This is also in agreement with the observation that the Asp122Asn substitution shows almost no effect on the turnover number (*k*_cat_) compared to wild-type (Table 1), making it likely that a backup mechanism is present. As shown in Figure S15, this mechanism is in good agreement with both the crystal structure (in terms of substrate positioning), and with the experimental activation free energy of 16.2 kcal mol^–1^ (based on the kinetic data presented in Table 1).

The final potential mechanism considered is a concerted mechanism (Figure 1D) involving intramolecular proton transfer from the attacking nucleophile, which is an active site water molecule. Although this pathway is less energetically unfavorable than the bridging hydroxide mechanism, it is significantly higher in energy than either the terminal hydroxide or Asp mechanisms, although mutations, in particular Asp122Asn (which eliminates the aspartic acid necessary for the Asp mechanism as well as the corresponding electrostatic repulsion between this side chain and the hydroxide nucleophile), could render this a viable pathway. However, despite the high energies of the bridging hydroxide and concerted pathways, both the terminal hydroxide and Asp mechanisms are energetically plausible, suggesting the presence of a catalytic backup, as observed in PON1^4^ and archaeal protein tyrosine phosphatases.^57−59^ Representative structures of key stationary points for each pathway are shown in Figures 3, S13, S14, and S16.

Overall, our calculations of the hydrolysis of the C6-HSL rule out the bridging hydroxide mechanism (Figure 1A) as an energetically viable mechanism, and similarly suggest a high barrier for the concerted mechanism (Figure 1D). In contrast, the terminal hydroxide (Figure 1B) and Asp (Figure 1C) mechanisms are shown to be similar in energy and competing pathways for the hydrolysis of this lactone.

### Empirical Valence Bond Simulations of the Hydrolysis of a Range of *N*-Acyl Homoserine Lactones by GcL Wild-Type and Variants

**Figure 4:**
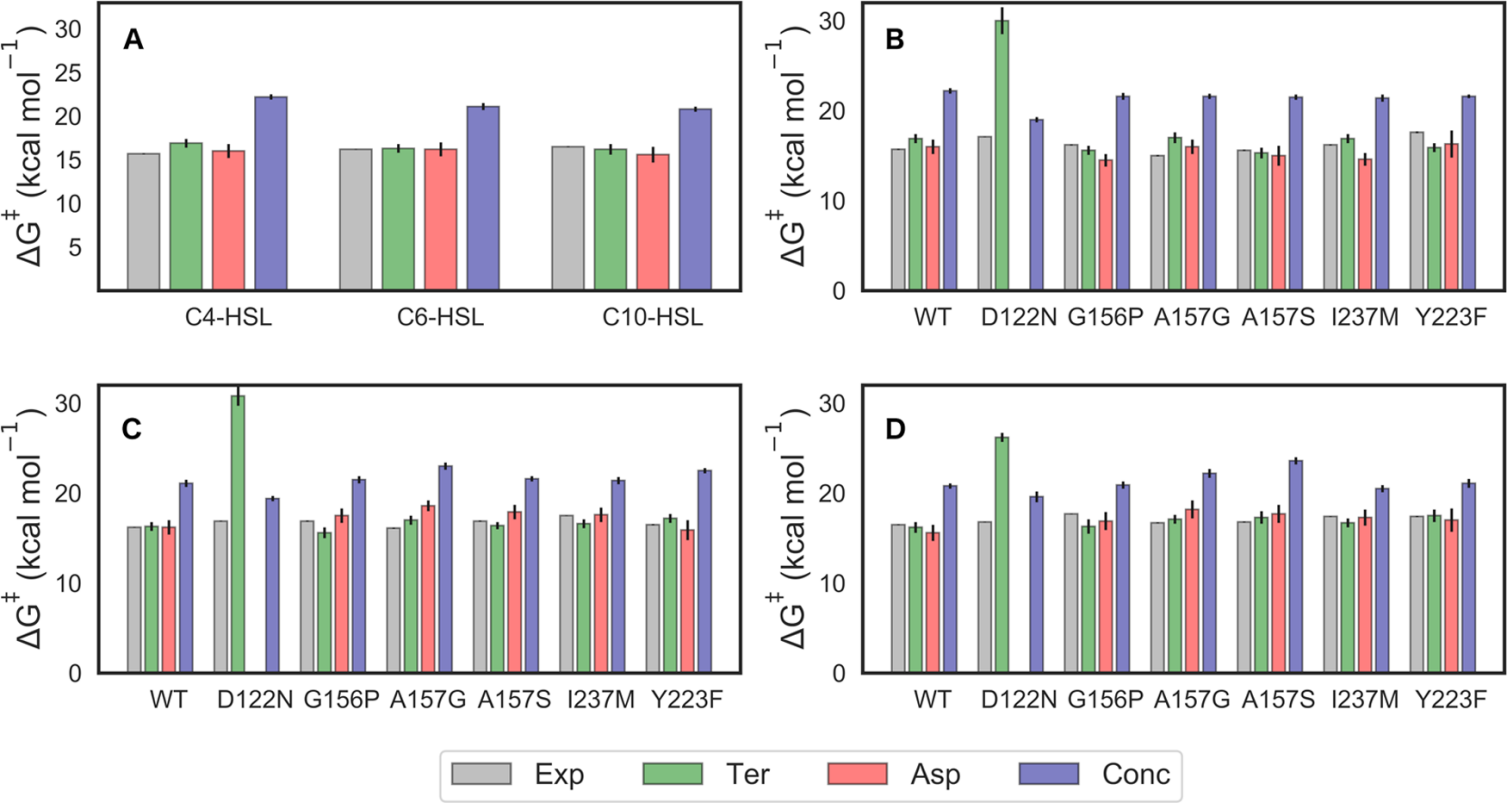
Comparison of the experimental (Δ*G*_exp_^‡^, gray) and calculated activation free energies for the terminal hydroxide (Ter), Asp, and concerted (Conc) mechanisms (Δ*G*_calc,_^‡^ green, salmon, and blue, respectively, see Figure 1B through D), for the hydrolysis of (A) a range of AHLs by wild-type GcL and (B, C, D) C4-, C6-, and C10-HSL, respectively, by wild-type GcL and the Asp122Asn, Gly156Pro, Ala157Gly, Ala157Ser, Tyr223Phe, and Ile237Met GcL variants. Error bars on the calculated values represent the standard error of the mean calculated over 30 discrete EVB trajectories for each system. The corresponding calculated data are shown in Tables 2 and S5–S8. The Δ*G*_exp_^‡^ values and their associated error bars were derived from the kinetic data (*k*_cat_ values) shown in Table 1 for each system.

To further explore the viability of the backup mechanisms across multiple substrates and enzyme variants, we performed additional EVB simulations of the hydrolysis of the C4-, C6-, and C10-HSLs (Figure S17) by wild-type GcL, as well as by the Asp122Asn, Gly156Pro, Ala157Gly, Ala157Ser, Tyr223Phe and Ile237Met GcL variants, following experimental data presented in Table 1. As the bridging hydroxide mechanism appears not to be energetically viable (Table 2), we focus here on modeling lactone hydrolysis proceeding through the terminal hydroxide, Asp, and concerted mechanisms (Figure 1B through D). The resulting data are shown in Tables S5–S8, and a comparison of experimental and calculated activation free energies is shown in Figure 4.

From these data, we see that both the terminal hydroxide and Asp mechanisms appear to be energetically accessible for all substrates and variants except Asp122Asn, with calculated activation free energies within ∼2 kcal mol^–1^ of both the experimental data and each other (Figure 4 and Table S8). Note that for the terminal hydroxide mechanism the first nucleophilic attack step is rate-limiting, while for the Asp mechanism the breakdown of the tetrahedral intermediate is rate-limiting, as shown in Tables S5 and S6. The pH rate dependency of the wild-type GcL and the variants further illustrate the enzyme’s catalytic redundancy (Figure S18). Indeed, with paraoxon, a promiscuous substrate with a good leaving group (paranitrophenolate), wild-type GcL and variants show higher activity levels with an increase of pH, within the tested range (6–10.5). The pH rate dependency of the lactonase activity, as reported by activity against the lactone TBBL, confirms the catalytic redundancy of GcL, yet does not allow to distinguish between possible mechanisms (Figure S18B). Specifically, In the case of TBBL (Figure S18A), we observe complex pH rate dependency, with the removal of key residues having minimal impact on the pH rate profile except in the case of the Y223F variant, where the pH rate profile becomes comparatively flat. This suggests both the presence of catalytic backups (making up for residue substitutions) and a putative role for Y223 in protonating the TBBL leaving group. In the case of paraoxon (Figure S18B), our measurements show a clear increase in activity with pH, which is most pronounced in the case of the Y223F variant, in contrast to TBBL, and consistent with the good *p*-nitrophenyl leaving group of paraoxon not necessarily needing protonation. We note that our EVB simulations (Table S8) already give reasonable agreement with experiment without the need for inclusion of proton transfer from Y223 to the lactone leaving group; this does not, however, rule out the possibility that such proton transfer would further enhance the reaction rate. Overall, while our pH rate profiles do not necessarily allow for direct mechanistic disambiguation, it is clear from this data that the pH dependency is complex and shifts subtly with variant, which would not be inconsistent with multiple mechanisms being at play, in particular given that the removal of key residues (with the exception of Y223) has little effect on the measured pH rate profiles.

In the case of the Asp122Asn variant, the Asp mechanism is no longer accessible due to the mutation of Asp122, leaving only the terminal hydroxide or concerted mechanisms as potential options. Curiously, in this variant, the activation free energy for the terminal hydroxide mechanism increases substantially, leaving the concerted mechanism (Figure 1D) as the only energetically plausible pathway with calculated activation free energies within 3 kcal mol^–1^ of the experimental data. Note that while this is still higher than the experimental values, the energy difference between calculated and experimental values is also smaller than that for other substrates/GcL variants, where the concerted pathway can be substantially higher in energy than experimental values (Figure 4 and Table S8). Visual examination of our EVB trajectories indicates that during our simulations, the substituted N122 side chain interacts with and stabilizes the terminal hydroxide ion at the Michaelis complex, contributing to higher calculated activation free energies through reactant state stabilization.

In summary, an EVB comparison of hydrolysis of different HSL substrates by wild-type GcL and variants indicates that the energetically preferred mechanism shifts depending on both substrate (tail length) and variant, suggesting that multiple mechanisms are plausible within the same enzyme active site, and that the selected mechanism will depend on precise environmental conditions, similar to prior work on PON1.^4,5^

### Molecular Dynamics Simulations of Effect of Tail Length on *N*-Acyl Homoserine Lactone Binding to GcL

In contrast to other lactonases,^30,31^ GcL is a generalist enzyme and is highly proficient toward AHLs with both short and long acyl chains as well as γ-, δ-, ε- and whiskey lactones, with catalytic efficiencies (*k*_cat_/*K*_M_) in the range of 10^4^ to 10^7^ M^–1^ s^–1^.^36^ However, even in this generalist enzyme, both lactone tail length and substituents impact both *k*_cat_ and *k*_cat_/*K*_M_, with longer lactone tail lengths showing improvements in catalytic efficiency, but diminished turnover numbers (note that the associated energy differences are small, on the range of 1.5 kcal mol^–1^ or less, based on kinetic data shown in Table 1). Furthermore, the AHL with the shortest acyl chain, C4-HSL, displays *K*_M_ values that are substantially higher than its longer chain counterparts C6-, C8-, or C10-HSL, and this feature is conserved not only for the wild-type enzyme but also for most of the variants studied in this work (Table 1). Our EVB calculations (Figure 4 and Table S8) give results in good agreement with experimental values for individual substrates but are unable to reproduce these rankings as the experimental differences in activation free energy are extremely small (1 kcal/mol or less) and beyond the resolution of current computational approaches. Therefore, to better understand the drivers of selectivity between different HSL substrates, we performed molecular dynamics simulations to explore the differences in structural stability of these substrates in the active site pocket of GcL as well as the binding modes of the lactone tail.

**Figure 5:**
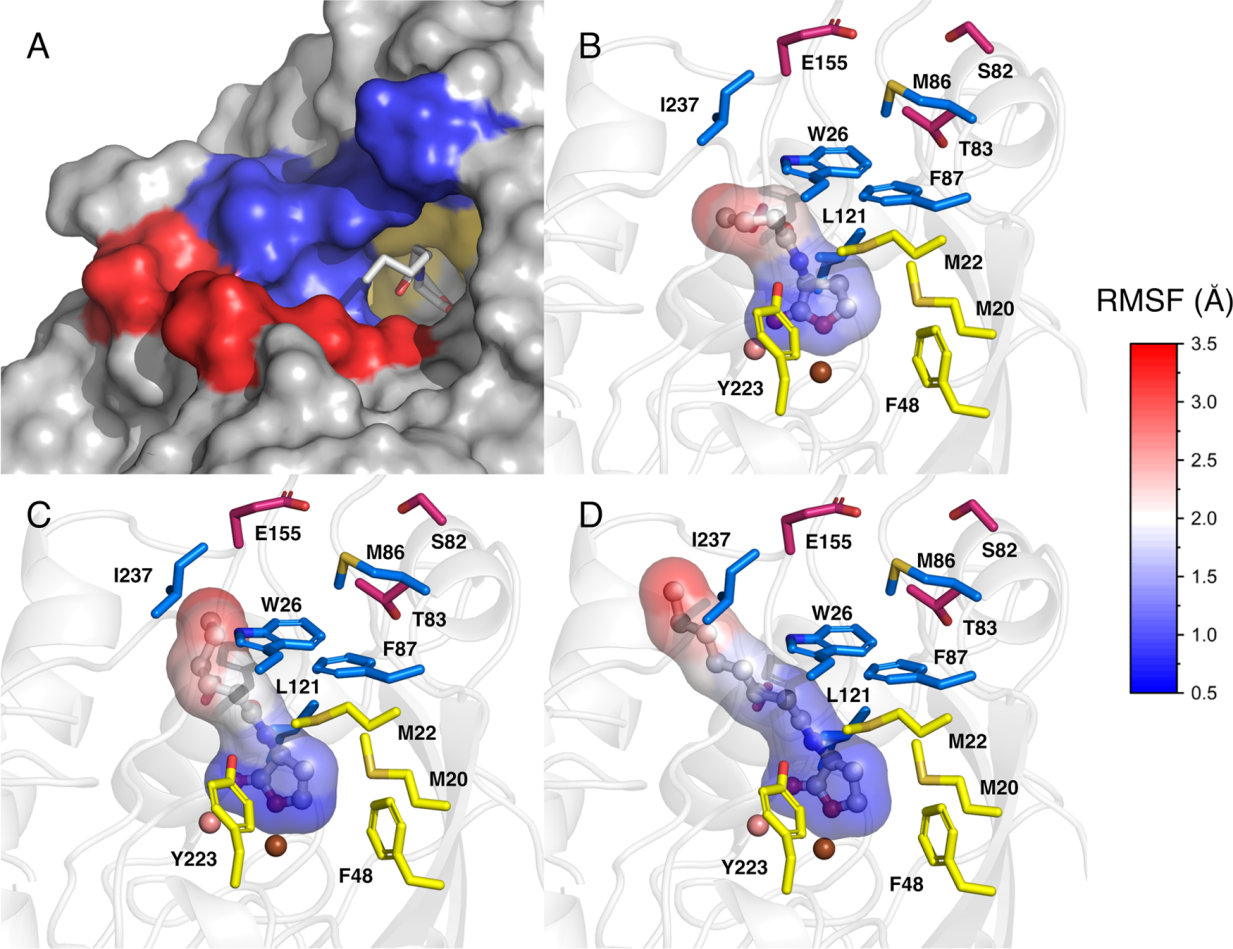
Root-mean-squared fluctuations (Å) of the heavy atoms of the C4-, C6-, and C8-HSL substrates during molecular dynamics simulations of wild-type GcL. Shown here are (A) a view of the active site pocket entrance of GcL in complex with C6-HSL, with the first and second hydrophobic patches shown in yellow and blue, respectively, and the hydrophilic region is shown in red. (B, C, D) Close-ups of the positions of the (B) C4-, (C) C6-, and (D) C8-HSL substrates in the GcL active site, colored by the root-mean-square fluctuations (RMSF) of the heavy atoms, to indicate substrate flexibility in the pocket. The side chains of the residues comprising the first and second hydrophobic patches are colored yellow and blue, respectively, and those comprising the hydrophilic patch are shown in mauve.

Structurally, the GcL active site comprises three subsites:^36^ a hydrophobic subsite (comprised of the side chains of Met20, Met22, Phe48 and Tyr223) involved in the accommodation of the lactone ring, a second hydrophobic patch (comprised of the side chains of Trp26, Met86, Phe87, Leu121 and Ile237) that accommodates the amide group and the beginning of the *N-*acyl chain of the substrate, and a hydrophilic region (comprised of the side chains of Ser82, Thr83, Glu155, Gly156 and Ala157) that is open to the protein surface and exposed to bulk water (Figure 5).

To shed light on how different tail lengths may affect the way different AHL substrates interact with the GcL active site, we performed molecular dynamics simulations of wild-type GcL in complex with C4-, C6-, and C8-HSL, as described in the Materials and Methods. The root-mean-square fluctuations (RMSF) of the heavy atoms of each substrate during these simulations show that the *N-*acyl chain of the substrate is highly mobile, positioning itself onto different pockets on the protein surface, as illustrated in Figures 5 and S19. In contrast, the metal-coordinating lactone ring is relatively rigid overall, although the shorter the alkyl tail, the greater (subtly) the flexibility of the ring (Figure 5). This flexibility of the tail may in turn render substrate stabilization through interactions with the second hydrophobic subsite.^36^ This is offset to some extent however by the fact that, based on our simulations, the shorter-chain C4-HSL substrate can bend its acyl tail to fit inside the subsite binding the lactone ring itself, resulting in the slightly higher *K*_M_ value observed for this substrate (Table 1).

Following this, when considering the locations of the amino acid substitutions performed in the variants studied here, two of them (Tyr223Phe and Asp122Asn) are part of the first hydrophobic subsite where the lactone ring is accommodated, Ile237Met is located at the second hydrophobic subsite, and the rest (Gly156Pro, Ala157Gly and Ala157Ser) are located in the third hydrophilic subsite. Especially noteworthy in Table 1 is the huge increase in the *K*_M_ values toward all the studied AHLs when the polar −OH group of Tyr223 is removed. The hydroxyl group of Tyr223 is hydrogen bonded to the carbonyl oxygen atom of the lactone ring in the crystal structure (PDB ID: 6N9Q(36) and 9AYT). Simulations of wild-type GcL in complex with C4-, C6- and C8-HSL (Table S9) indicate the presence of an interaction between the OH group of the Tyr223 side chain and either the amine nitrogen or the carbonyl oxygen of the alkyl tail for at least 22% of the simulation time (this is most pronounced in simulations with C6-HSL). Our simulations indicate that this is either a direct interaction between the tyrosine side chain and the lactone tail or a water-mediated interaction with a bridging water molecule (present for an additional ∼10% of simulation time). As this interaction contributes to the stability of the lactone in the active site pocket, its elimination in the Tyr223Phe variant clearly results in the corresponding *K*_M_ values of its substrates as well as its catalytic activity.

**Figure 6:**
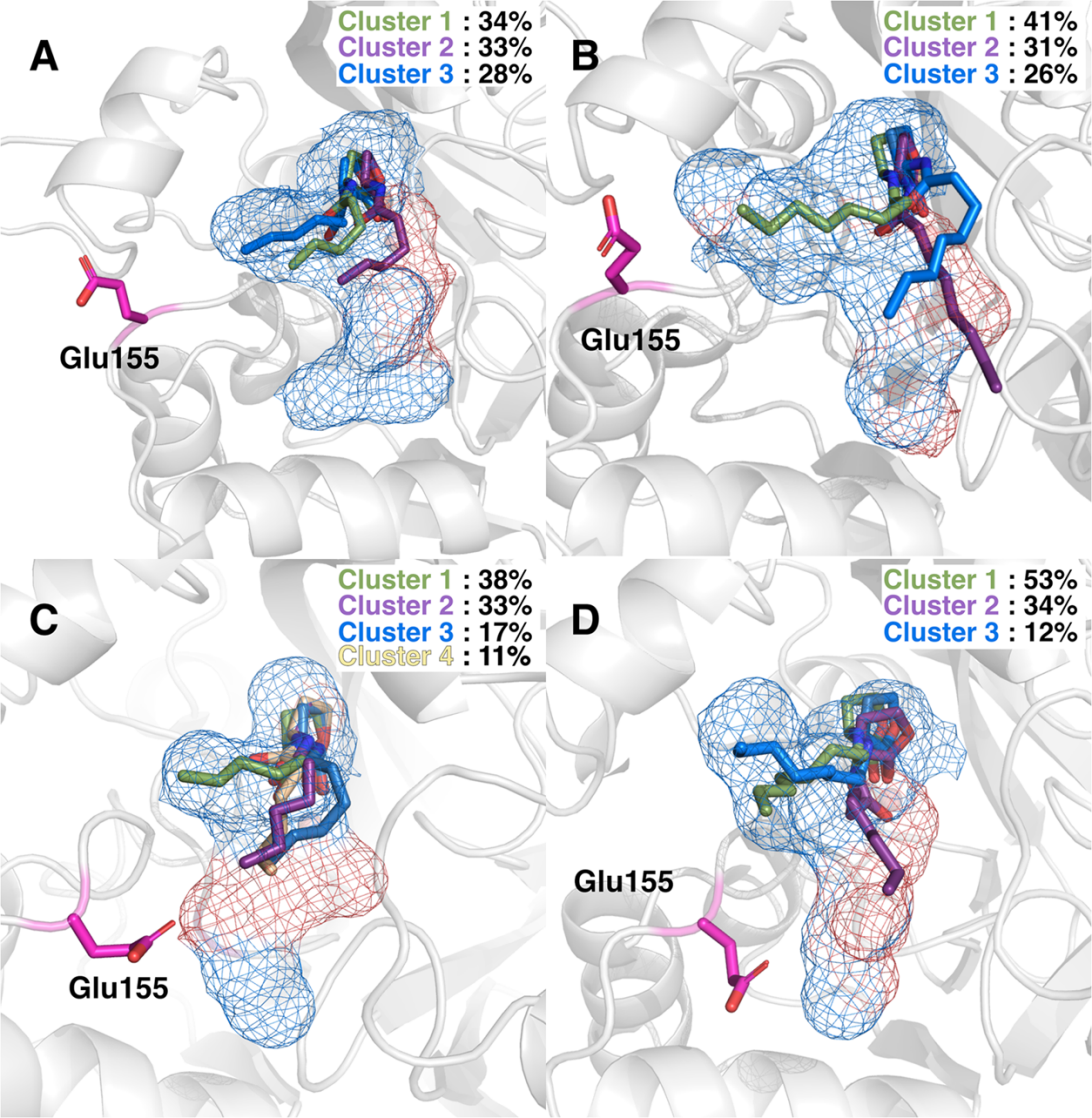
Main conformation of the binding pocket from RMSD clustering of our MD simulations of (A, C) C6- and (B, D) C8-HSL, in complex with (A, B) wild-type GcL and (C, D) the Gly156Pro variant, The binding pocket is shown as a blue grid, with the hydrophilic regions colored in red. Substrate structures from the three/four principal clusters of each system (obtained from RMSD clustering) in complex with C6- or C8-HSL, as well as the dominant position of the Glu155 side chain, are highlighted. Only substrate structures from clusters accounting for more than 10% of the simulation time are shown here.

Furthermore, while most of the amino acid substitutions summarized in Table 1 do not lead to major structural changes (based on structural data), the crystal structure of the Gly156Pro variant (PDB ID: 9B2I) reveals structural rearrangement of the Asn152-Ala157 loop, such that the polar residue Glu115 is relocated from pointing out of the binding pocket to pointing into the binding cleft. When the effect of this mutation on the reaction kinetics of the different substrates is examined, it is surprising to see a large increase in the *K*_M_ value of the C6-HSL substrate, while the *K*_M_ of C8-HSL remains similar to the wild-type. We used MDpocket^70^ to locate the hydrophobic/hydrophilic regions of the cavity along with the MD simulations of C6-, and C8-HSL in complex with wild-type GcL and Gly156Pro variant. Figure 6 shows representative structures from the main clusters, describing the most sampled populations along the simulations, of each substrate in the main representative binding pocket of the relevant GcL variant (obtained from RMSD clustering across our simulations, as described in Materials and Methods), with the corresponding hydrophilic regions colored in red. Interestingly, the end of the C6-HSL acyl chain, which is the most mobile part of the substrate, lays in the same position as the rearranged residue Glu155 side chain, creating strong repulsion and destabilizing the substrate. In contrast, the end of the C8-HSL acyl chain lies further from the active site and the repulsion between Glu155 and the substrate tail is diminished, allowing tighter binding of C8- compared to C6-HSL.

Additionally, these data illustrate significant conformational flexibility of the alkyl tail of both C6- and C8- substrates in both wild-type GcL and the Gly156Pro variant (Figure 6), highlighting the conformational heterogeneity of the substrate in the active sites and the fact that it can accommodate multiple binding poses of the alkyl tail. This effect is significantly more pronounced in the complex of Gly156Pro with C8-HSL than that with C6-HSL, suggesting that part of the reason for its better *K*_M_ value for C8-HSL is simply its ability to sit in multiple binding modes in the active site pocket, avoiding Glu155. This flexibility in binding mode will also affect how different AHL substrates interact with different GcL variants, as amino acid substitutions reshape the active site pocket. When this is coupled with the ability of short-chain HSLs such as C4-HSL to explore and occupy new binding pockets (Figure 5), this conformational plasticity will impact both activity and substrate specificity, as shown in Table 1.

Finally, when considering the effects on activity of the Ala157Gly, Ala157Ser, Gly156Pro, and Ile237Met variants (Table 1), we observe that these variants show dramatically altered kinetics for only some specific substrates, resulting in changes in substrate preference. This leads us to question whether these mutations are altering the active site structure in comparison to wild-type GcL. To address this, we expanded our molecular dynamics simulations to cover all four variants, performing simulations of each variant in complex with each of the C4-, C6- and C8-HSL substrates (see the Materials and Methods). Analysis of these trajectories show that both the overall protein structure (Figure S20) and the active site structure (Figure S21) are stable on the simulation time scale (3 × 500 ns simulations per system), and any structural changes observed here are subtle. Thus, the change in substrate selectivity is less likely to be due to a radical structural rearrangement of the active site. Rather, this could stem from the additive effect of multiple subtle shifts in structural parameters, such as active site solvation and the precise positioning of key active site residues, which also reflects the overall modest changes in lactonase activity compared to wild-type.

### Empirical Valence Bond Simulations of Lactone Hydrolysis by Other Metallo-β-lactamase-like Lactonases

Our EVB simulations support the existence of catalytic redundancies in GcL and provide a molecular rationale for this redundancy as well as the associated substrate specificity toward different AHL substrates. Such catalytic backups have been previously suggested in the case of an analogous lactone, PON1.^4^ Furthermore, several archaeal protein tyrosine phosphatases have been suggested to operate via dual general acid mechanisms, with built-in redundancies in the active site.^57−59^ As GcL and PON1 (let alone protein tyrosine phosphatases) have rather different active site architectures, particularly in terms of the identity and coordination of the metal centers involved, this then raises the question of whether catalytic redundancies and backups are a common feature of promiscuous lactonases (and enzymes more broadly).

To address this in the context of lactonases, we extended our EVB simulations to two additional MLLs: AiiA and AaL. These systems were selected because of the structural similarity of the corresponding binding domains, the availability of high resolution crystal structures,^34,56^ and the availability of kinetic data for these enzymes against C6-HSL,^27,55^ which allow us to compare them directly to our GcL simulations (Table 2). The overall structure of AaL is very similar to that of GcL, with an RMSD of 0.42 Å (sequence identity 81.1%). There are larger differences between AiiA and GcL, with an RMSD of 1.22 Å between the two structures (24.4% sequence identity). AiiA also lacks a protruding loop involved in dimerization in GcL and AaL,^34,36^ and is instead organized as a monomer.^71^ The different key regions of the AHL binding pocket are highly conserved among the three MLLs, and Tyr223 is conserved in all MLLs except one known example (AidC, Figure S22(32)).

We extended our EVB simulations of all studied mechanisms to the AiiA and AaL lactonases in complex with C6-HSL using the same set of parameters as in GcL (Table 3). As in wild-type GcL (Tables 2 and S8), the bridging hydroxide and concerted mechanisms (Figures 1A,D) yield activation free energies that are too high and are therefore unlikely, while both the terminal hydroxide and Asp mechanisms (Figure 1B,C) are energetically feasible and within the range of the experimental data. We note the slightly lower calculated activation free energies for the bridging hydroxide mechanism for all enzymes studied; however, this could be the same underestimation of the activation free energy for this mechanism for the hydrolysis of the C6-HSL substrate, as in the case of GcL. Based on this data, we demonstrate that the mechanistic plasticity of GcL is conserved across these three diverse lactonases and is not a feature unique to GcL.

**Table 3:** Comparison of Experimental and Calculated Activation Free Energies (kcal mol^–1^) for the Hydrolysis of C6-HSL Catalyzed by Three Wild-Type MLLs, GcL, AiiA, and AaL, through Different Mechanisms Considered in this Work^a^

	GcL	AiiA	AaL
Experimental	16.2	14.8	15.9
Bridging Hydroxide Mechanism	36.7 ± 1.1	25.6 ± 0.4	29.7 ± 0.4
Terminal Hydroxide Mechanism	16.3 ± 0.5	15.5 ± 0.7	14.9 ± 0.8
Asp Mechanism	16.2 ± 0.8	13.8 ± 0.3	14.4 ± 0.7
Concerted Mechanism	21.1 ± 0.4	18.4 ± 0.3	25.4 ± 0.9

a The different mechanisms considered here are illustrated in Figure 1. All values shown here are averages and standard error of the mean over 30 independent EVB trajectories, shown in kcal mol^–1^. Experimental (exp) activation free energies for the hydrolysis of C6-HSL by GcL, AiiA, and AaL are obtained based on kinetic data provided in refs (27 and 55). (Table S10).

## Overview and Conclusions

Lactones have a wide range of biological activities,^72^ including acting as antimicrobial agents,^73^ anti-inflammatory compounds,^74^ antitumor agents,^75,76^ and mycotoxins,^77^ and are abundant in cellular metabolism.^78^ The importance of lactones to bacterial communication (*QS*)^79^ and biofilm formation^13^ makes the enzymes that degrade them biotechnologically important as *QQ* agents for a host of industrial and biomedical applications. Because lactones play such diverse roles in biology, the true primary purpose of many lactonases remains unclear: for example, the first identified lactonase AiiA^71,80^ exhibits millimolar *K*_M_ values toward short AHL substrates and is a broad generalist, with very little discrimination between substrates regardless of varying chain lengths and/or substitutions on the chain.^27,30,56,81^ It is therefore unclear whether AiiA evolved specifically for the purpose of quenching microbial signaling: it is clearly capable of doing so but likely has a much broader biological purpose than *QQ* alone. PON1 is also a lactonase/organophosphate hydrolase^20^ that acts as a broad scavenger enzyme. In contrast, GcL, the primary focus of this study, remains a generalist enzyme but with lower micromolar *K*_M_ values, making it perhaps more likely specialized for *QQ* as its native function.

We examined the mechanism and substrate selectivity of wild-type GcL and two related lactonases, AiiA and AaL (sequence similarity to GcL = 41.5 and 89.2%, respectively), through a combination of structural, biochemical, and computational approaches. Our structural and mutagenesis analyses highlight important roles for Asp122 and Tyr223 in catalysis and substrate positioning, yet neither residue seems completely necessary for catalysis. Remarkably, our mechanistic analyses indicate that there are not one but two viable (and energetically similar) mechanisms for AHL hydrolysis by GcL with the preferred mechanism between the two mechanisms shifting, depending on both different AHL substrates and different enzyme variants. This is complemented by substrate plasticity in the active site, with the alkyl tail of the AHL substrates taking on multiple conformations depending on the tail length and enzyme variant. This mechanistic redundancy is observed again in our simulations of both AiiA, and AaL as well as in computational and experimental studies of other enzymes such as PON1^4,5^ and archaeal protein tyrosine phosphatases.^57−59^

The importance of conformational dynamics to enzyme selectivity and evolvability is by now well established.^82−89^ Our data indicate that, similar to catalytic promiscuity and broad substrate specificity, mechanistic promiscuity also plays an important role in modulating enzyme activity and selectivity. To put into context, our prior work on PON1 has shown that PON1 is not only mechanistically promiscuous,^4^ but it is also possible to control the catalytic mechanism of PON1 toward a given substrate by (1) mutating a key catalytic residue essential for the primary catalytic mechanism, and (2) laboratory evolution experiments to optimize activity through the backup mechanism.^5^ In this work, we show that such promiscuity is not unique to PON1 but is also observed in a range of lactonases and can likely be similarly exploited in an engineering effort to control the substrate preference of this enzyme. Taken together, these data expand the question from “how does a promiscuous enzyme chooses a specific substrate from a pool of different substrates?” to also “how does the enzyme utilize a specific mechanism from a pool of different mechanisms?” and “what makes an active site catalytically versatile?” This is a broader issue that requires examination, as it is now observed across an increasing number of systems and should be a significant consideration when engineering generalist enzymes for more specific functions.

## Materials and Methods

### Mutagenesis

Site-directed mutagenesis was performed for the mutations Asp122Asn and Tyr223Phe using Pfu polymerase (Invitrogen) on 100 ng of plasmid using primers (Table S11), with an annealing temperature of 63 °C for 34 cycles. After DpnI digestion, plasmids were concentrated by ethanol precipitation and then transformed (Gene-Pulser, Bio-Rad) into *Escherichia coli* cells DH5α (Invitrogen) by 30 s of heat shock at 42 °C.

Additional mutations of key positions of GcL identified from the previous structural analysis of GcL^36^ were ordered from Genscript Biotech Corporation (catalog SC2029) as part of saturation mutagenesis libraries designed to optimize GcL properties (results from this engineering efforts will be reported in detail in a separate work). 100 ng of the pooled plasmid library was introduced into *E. coli* DH5α by heat shock transformation at 42 °C, and the cells were spread onto LB agar supplemented with ampicillin. Individual colonies were resuspended in phosphate-buffered saline and sent to ACGT Inc. for direct colony sequencing. Identified single mutant plasmids were expressed in *E. coli* DH5α and purified using a Qiagen miniprep kit. Purified mutant plasmids were confirmed by sequencing (University of Minnesota Genomics Center) before finally being transformed into an *E. coli* protein production strain by heat shock.

### Protein Production

The various proteins were produced in the *Escherichia coli* strain BL21(DE3)-pGro7/GroEL strain (TaKaRa). The sequences were enhanced by an N-terminal strep tag (WSHPQFEK) with a TEV cleavage site sequence (ENLYFQS). Protein productions were performed at 37 °C in the autoinducer media ZYP (with 100 mg·ml^–1^ ampicillin and 34 mg·ml^–1^ chloramphenicol). When cells reached the exponential growth phase, the chaperone GroEL was induced by adding 0.2% l-arabinose, 2 mM CoCl_2_ was added, and the cultures were transitioned to 18 °C for 16 h. Cells were harvested by centrifugation (4400 *g*, 4 min, 4 °C), resuspended in lysis buffer (150 mM NaCl, 50 mM HEPES pH 8.0, 0.2 mM CoCl_2_, 0.1 mM PMSF and 25 mg mL^–1^ lysozyme), and left on ice for 30 min. Cells were then sonicated (amplitude 45% in three steps of 30 s; 1 pulse-on; 2 pulse-off) (Q700 Sonicator, Qsonica). Cell debris was removed by centrifugation (5000*g*, 45 min, 4 °C).

### Protein Purification

The lysis supernatant was loaded on a Strep Trap HP chromatography column (GE Healthcare) in PTE buffer consisting of 50 mM HEPES pH 8.0, 150 mM NaCl, and 0.2 mM CoCl_2_ at room temperature. TEV cleavage was performed by adding the Tobacco Etch Virus protease (TEV, reaction 1/20, w/w) overnight at 4 °C. Then, the sample was loaded on a size exclusion column (Superdex 75 16/60, GE Healthcare) to obtain pure protein. Sodium dodecyl sulfate polyacrylamide gel electrophoresis (SDS-PAGE) was performed to confirm the identity and purity of the proteins. Proteins were quantified by measuring their absorbance at 280 nm and using the Beer–Lambert law. The protein molecular extinction coefficient was generated using the protein primary sequence and the ProtParam tool implemented into ExPASy.^90^

### Determination of Kinetic Parameters

All kinetic experiments were performed in at least triplicates in 200 μL reaction volumes using 96-well plates (6.2 mm path length cell) and a microplate reader (Synergy HT), using the Gen5.1 software at 25 °C. The time course of the hydrolysis of AHL substrates was analyzed by monitoring the decrease in absorbance at 577 nm. Lactone hydrolysis assays were performed in lactonase buffer (2.5 mM bicine, pH 8.3, 150 mM NaCl, 0.2 mM CoCl_2_, 0.25 mM *m*-cresol purple, and 0.5% dimethyl sulfoxide (DMSO)), using *m*-cresol purple (p*K*_a_ 8.3 at 25 °C) as a pH indicator to follow the acidification caused by lactone ring hydrolysis. The molar extinction coefficient was measured by recording the absorbance of the buffer over a range of acetic acid concentrations between 0–0.35 mM. The initial rates of reactions were fitted using GraphPad Prism 5 for Windows (GraphPad Software, San Diego, California) and fitted to a Michaelis–Menten curve to obtain the catalytic parameters. Due to the limits in the sensitivity of this pH indicator-based assay at low substrate concentration, measurements for substrate with very low *K*_M_ values are challenging and can result in poorer fit to the Michaelis–Menten equation. Substrate/enzyme variant combinations with very high *K*_M_ values are fit to a linear regression due to limits in substrate solubility. Replicates with technical errors (e.g., pipetting errors or failed) were excluded from the Michaelis–Menten analysis.

The catalytic activity of the enzymes against paraoxon ethyl was monitored using a previously described assay.^36^ The reaction was monitored by following the production of paranitrophenolate anions at 405 nm. Reactions were performed in 50 mM HEPES pH 8.0, 150 mM NaCl, and 200 mM CoCl_2_ using ε_405nm_ = 17,000 M^–1^ cm^–1^.

The specific activity of GcL against paraoxon ethyl at different pHs were measured in 200 μL reactions containing 1 mM paraoxon ethyl, 5 μg enzyme and 50 μM CoCl_2_ in 50 mM buffers. The buffers used were 50 mM MES pH 6, 6.5; 50 mM HEPES pH 7, 7.5, 8; CHES pH 8.6, 9, 9.5, 10; and CAPS pH 10.5. The change in absorbance was measured at 412 nm, and the extinction coefficient of 4-nitrophenol (18,300 M^–1^ cm^–1^) was used to calculate the specific activity. Reactions were performed in triplicate. The specific activity of GcL against thiobutyl butyrolactone^91^ (TBBL) was measured at different pHs in 200 μL reactions containing 1 mM TBBL, 5 μg enzyme, 50 μM CoCl_2_, 2 mM 5,5′-dithiobis(2-nitrobenzoic acid) (DTNB) in 50 mM buffers as outlined in paraoxonase activity. Single reactions were conducted due to a limiting amount of substrate. The change in absorbance was measured at 412 nm and the specific activity was calculated using the extinction coefficient of TNB (14,150 M^–1^ cm^–1^).

### Crystallization, Data Collection, and Refinement

Crystallization of GcL wild-type and variants was performed using protein samples concentrated to 10.0–11.5 mg mL^–1^ using the hanging-drop vapor-diffusion method and previously reported conditions.^36^ The best crystals were produced with 1.0–1.25 M ammonium sulfate and 0.1 M sodium acetate, pH 4.0–5.5. The structure in complex with the substrate C6-HSL was obtained by soaking the crystals for 10 min in a solution containing the cryoprotectant and 20 mM of C6-HSL. The GcL complex with the reaction product of C8-HSL was obtained by cocrystallizing the enzyme with 20 mM C8-HSL (final). The crystals were cryoprotected in a solution composed of 30% PEG 400 and frozen in liquid nitrogen. X-ray diffraction data sets were collected at 100 K using synchrotron radiation on the 23-ID-B and 23-ID-D beamlines at the Advanced Photon Source (APS, Argonne, Illinois, Table S1). The structures were resolved in the H3 space group for the Asp122Asn-2metals and the structure bound to C6-HSL, and in C2 for the other structures. The integration and the scaling of the X-ray diffraction data were performed using the XDS package.^92^ The molecular replacement was performed using the wild-type GcL structure as a model (PDB ID: 6N9I(36)) and using MOLREP.^93^ Manual model construction was performed with Coot.^94^ Cycles of refinement were performed using REFMAC.^95^ Statistics are listed in Table S1. We note that the ligand occupancy in the structures is variable in the different monomers that are present in the asymmetric units, and the highest occupancy models (presented) are 0.8 for the C6-HSL and 0.7 for the C8-HSL product. These occupancy levels limit the accuracy of the models.

### Empirical Valence Bond Simulations

The empirical valence bond (EVB) approach^54^ is a force field-based approach that describes chemical reactivity within a valence bond-based quantum mechanical framework. This approach has been used extensively to describe enzyme reactivity in general,^96,97^ and lactone hydrolysis in particular.^5,61,62^ In this work, we have modeled the hydrolysis of the C4-, C6- and C10-HSL (Figure S17) by wild-type AiiA, AaL and GcL, as well as the Asp122Asn, Gly156Pro, Ala157Gly, Ala157Ser, Tyr223Phe, and Ile237Met GcL variants, through four different mechanisms shown in Figure 1. All four mechanisms were tested for the hydrolysis of C6-HSL by wild-type GcL, and the energetically accessible pathways, i.e., the terminal hydroxide, Asp, were tested for the hydrolysis of all other compounds. The corresponding valence bond states are shown in Figure S23. Note that the bridging and terminal hydroxide mechanisms shown in Figure 1 use identical valence bond states for the first step of the reaction, as the only difference between them is whether the nucleophile is in the metal coordination of the hydroxide ion. In addition, the first three mechanisms considered in Figure 1 are all 3-state stepwise processes, whereas the final mechanism is a 2-state concerted process. In the case of the stepwise processes, the intermediate state structures generated at the end point of EVB simulations of the first step for each replica were used as starting points for EVB simulations of the second step of the reaction.

All relevant input and parameter files necessary to reproduce our calculations as well as snapshots from our simulation trajectories have been uploaded as a data package to Zenodo, DOI: 10.5281/zenodo.11072674. Full details of system setup and EVB simulations, are provided in the Supporting Information and summarized here in brief. All EVB simulations were performed using the *Q5* simulation package^98^ and the OPLS-AA force field,^99^ as implemented into *Q5*. Metal and ligand parametrization, and calibration of the EVB off-diagonal term and gas-phase shift, were performed as described in the Supporting Information. The same parameter set was then transferred unchanged in simulations of each substrate with all enzyme variants, as the EVB off-diagonal term has been shown to be phase-independent and thus transferable.^100,101^

Simulations of wild-type AaL were performed using the structure of wild-type AaL in complex with C6-HSL, obtained from the Protein Data Bank^102^ (PDB ID: 6CGZ(34)), while for wild-type AiiA simulations, the structure of AiiA in complex with C6-HSL hydrolytic product was used (PDB ID: 3DHB(56)), where the product was replaced with C6-HSL by aligning AiiA and AaL wild-type structures. Due to the high negative charge of the system that fits inside the water droplet in the case of AiiA, 10 Na^+^ counterions were added to neutralize the system. These counterions were placed so that they interact with negatively charged residues near the surface of the enzyme that still fall within the water droplet. All simulations of wild-type GcL were performed using the structure of wild-type GcL in complex with C4-HSL and C6-HSL (PDB IDs: 6N9Q(36) and 9AYT). The structure of the Tyr223Phe variant was generated by manual deletion of the Tyr223-OH group from the wild-type structure, while the Asp122Asn, Gly156Pro, Ala157Gly, Ala157Ser and Ile237Met substitutions were introduced by use of the Dunbrack 2010 Rotamer Library,^103^ as implemented in USCF Chimera, v. 1.14.,^104^ trying to reproduce as much as possible the rotamer found in the crystal structures where unliganded structures including those substitutions were available (PDB IDs: 9B2L, 9B2I and 9B2J for the Asp122Asn, Gly156Pro, and Ile237Met variants, respectively). Unliganded structures were not used directly for these simulations since the loop comprising residues 236–238 is found in a closed conformation in the unliganded structures, creating a steric clash with the substrate tail when aligned with the liganded complex; wild-type liganded complexes, by contrast, suggest that this loop opens to provide more optimal substrate positioning.

All the variants were simulated in complex with the different substrates of interest to this work (Figure S17). In each case, starting structures for all Michaelis complexes with the substrates C4-, C6- and C10-HSL were either taken directly from the crystal structure (where a liganded complex was available), or generated manually based on an overlay with the coordinates for C4- or C6-HSL in the wild-type structure (PDB IDs: 6N9Q(36) and 9AYT, respectively).^36,102^ The system was then solvated in a 30 Å droplet of TIP3P water molecules,^105^ described using the surface constrained all-atom solvent (SCAAS) approach.^106^ The protonation states of all relevant ionizable residues, as well as histidine protonation patterns, are listed in Table S12. The starting structures for the terminal hydroxide mechanism were generated as described above but with rotation of the lactone ring to place an extra hydroxide ion on the Fe^2+^ metal center. (The starting structures used in our simulations can be found in DOI: 10.5281/zenodo.11072674).

All systems were gradually heated from 1 to 300 K, as described in the Supporting Information, followed by 50 ns of molecular dynamics equilibration at the target temperature, the convergence of which is shown in Figures S24–S27. Thirty individual replicas were generated per system using different random seeds to assign initial velocities. The end point of each equilibration was used as the starting point for 30 subsequent EVB simulations (1 EVB simulation per replica), which were performed using the valence bond states shown in Figure S23, using 51 EVB mapping windows of 200 ps/length each (i.e., 10.2 ns simulation time per EVB trajectory). This led to a cumulative total of 1.5 μs equilibration and 306 ns EVB sampling per system and mechanism (612 ns EVB sampling for the mechanisms comprising a three-state process) and a total of 154.6 μs simulation time (equilibration + EVB) over all systems.

All equilibration and EVB simulations were performed using the leapfrog integrator with a 1 fs time step, using the Berendsen thermostat^107^ to keep the temperature constant with a 100 fs bath coupling time, and with the solute and solvent coupled to individual heat baths. Long-range interactions were treated using the local reaction field (LRF)^108^ approach, while cut-offs of 10 and 99 Å were used for the calculation of nonbonded interactions involving the protein and water molecules and the EVB region respectively (effectively no cutoff for the latter). In all but the very initial minimization step to remove bad hydrogen contacts, the SHAKE^109^ algorithm was applied to constrain all bonds involving hydrogen atoms. Further simulation details, as well as details of simulation analysis, are provided in the Supporting Information.

### Molecular Dynamics Simulations

All molecular dynamics simulations were performed using the GROMACS v2021.3 simulation package,^110,111^ in combination with the OPLS-AA force field^99^ for compatibility with our EVB simulations. In this work, we have performed simulations of the wild-type GcL, as well as Gly156Pro, Ala157Gly, Ala157Ser, Ile237Met and Tyr223Phe variants, in complex with C4-, C6- and C8-HSL. The starting structure for our simulations was taken from crystallographic coordinates of wild-type GcL in complex with C4- and C6-HSL (PDB IDs: 6N9Q(36) and 9AYT). The histidine protonation patterns in our MD simulations were identical to those used in our EVB simulations, as listed in Table S12, and all other ionized residues (Asp, Glu, Arg, and Lys) were modeled in their standard ionization states under physiological conditions, i.e., Asp and Glu side chains were negatively charged while Arg and Lys side chains were positively charged. The resulting complex was put in the center of an octahedral box filled with TIP3P water molecules,^105^ with at least 10 Å distance between the surface of the complex and the edge of the box. Na^+^ ions were added to neutralize each system. After the system setup was complete, three independent replicas were generated, where a 5000-step minimization was performed on each system using the steepest descent and conjugate gradient methods, followed by heating of the solvated system from 0 to 300 K over a 500 ps MD simulation in an NVT ensemble, using the velocity rescaling thermostat^107,112^ with a time constant of 0.1 ps for the bath coupling. This was again followed by a further 500 ps of simulation in an NPT ensemble at 300 K and 1 bar, controlled by the same thermostat and a Parrinello–Rahman barostat^107^ with a time constant of 2.0 ps. Positional restraints of 2.4 kcal mol^–1^ Å^–2^ were applied on every heavy atom in each of the xyz directions for the first two steps of equilibration. Afterward, the positional restraints were released, and instead, distance restraints were applied between all the side chains coordinating the dummy particles and the metal centers, including the ligand, and the central atom of the dummy complex, during the first 25 ns of production to ensure crystallographic ligand coordination around the metal ions is maintained. These distance restraints were set to 40 kcal mol^–1^ Å^–2^ during the first 20 ns of simulation time, with the force constant halved to 20 kcal mol^–1^ Å^–2^ the last 5 ns of simulation time. Finally, 500 ns of unrestrained molecular dynamics simulations (x 3 replicas) were performed for each system, the convergence of which are shown in Figure S20. For all the simulations, 12 Å nonbonded interaction cutoff was used to evaluate long-range electrostatic interactions, using the Particle Mesh Ewald (PME) algorithm,^113^ and the LINCS algorithm^114^ was applied to constrain all hydrogen bonds, using a 1 fs time step.

### Simulation Analysis

The physicochemical properties of the active site pocket of the wild-type, Gly156Pro, and Tyr223Phe GcL enzymes when either C4-, C6- or C8-HSL is bound, were tracked along the corresponding conventional molecular dynamics simulations trajectories using the MDpocket^70^ tool, published within the fpocket^115^ suite of pocket detection programs. To account for the structural differences inferred by each ligand on the cavity, the ligand trajectories were used, where the corresponding ligand was stripped from the cavity to assess the pocket. All cavities were identified using a frequency isovalue of 0.7, and points corresponding to the active site pocket were selected and tracked throughout the trajectories.

All other analyses were performed using the CPPTRAJ^116^ module of the AmberTools19^117^ suite of programs. The most-populated structures were obtained by clustering together 3 independent 500 ns MD simulations for each system using a hierarchical algorithm and selecting the centroid of the top-ranked clusters. The clustering was performed based on pairwise RMSD calculations over all the atoms of the ligand. All hydrogen bonds formed between the AHL and Tyr223 were identified using a donor–acceptor distance cutoff of 3.5 Å, and a donor-hydrogen-acceptor angle of 135 ± 45°. Only hydrogen bonds with an occupancy of >1% of the cluster simulation time were considered. Root-mean-square fluctuations (RMSF, Å) of the heavy atoms of the ligand, were calculated over 3 independent 500 ns MD simulations for each system.

## Supporting Information Available

Additional computational methodology, simulation analysis, and kinetic data (PDF)
